# Butyl 4-(4-methyl­benzene­sulfonamido)­benzoate

**DOI:** 10.1107/S1600536812015413

**Published:** 2012-04-28

**Authors:** Ghulam Mustafa, Mehmet Akkurt, Yılmaz Dağdemir, Islam Ullah Khan

**Affiliations:** a Department of Chemistry, GC University, Lahore 54000, Pakistan; b Department of Physics, Faculty of Sciences, Erciyes University, 38039 Kayseri, Turkey

## Abstract

In the title compound, C_18_H_21_NO_4_S, the aromatic rings are almost normal to each other, with a dihedral angle of 89.27 (18)°. The mol­ecular conformation is stabilized by an intra­molecular C—H⋯O inter­action, which generates an *S*(6) motif. In the crystal, N—H⋯O and C—H⋯O hydrogen bonds lead to the formation of chains propagating along [010]. Neighbouring chains are linked *via* a C—H⋯π inter­action. The –CH_2_CH_2_CH_3_ atoms of the butyl group are disordered over two sets of sites, with a refined site-occupancy ratio of 0.536 (16):0.464 (16).

## Related literature

For related structures, see: Mustafa *et al.* (2010 ▶, 2011 ▶, 2012 ▶); Khan *et al.* (2011 ▶). For bond-length data, see: Allen *et al.* (1987 ▶). For the graph-set analysis of hydrogen bonding, see: Bernstein *et al.* (1995 ▶).
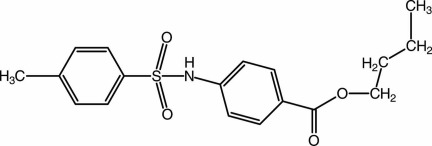


## Experimental

### 

#### Crystal data

C_18_H_21_NO_4_S
*M* *_r_* = 347.43
Monoclinic, 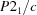

*a* = 17.8216 (13) Å
*b* = 8.2702 (6) Å
*c* = 11.9282 (8) Å
β = 91.001 (3)°
*V* = 1757.8 (2) Å^3^
*Z* = 4
Mo *K*α radiation
μ = 0.21 mm^−1^
*T* = 296 K
0.33 × 0.25 × 0.21 mm

#### Data collection

Bruker APEXII CCD diffractometer
13164 measured reflections
3557 independent reflections
2287 reflections with *I* > 2σ(*I*)
*R*_int_ = 0.038

#### Refinement

*R*[*F* ^2^ > 2σ(*F* ^2^)] = 0.072
*wR*(*F* ^2^) = 0.218
*S* = 1.05
3557 reflections
224 parameters
H-atom parameters constrained
Δρ_max_ = 0.47 e Å^−3^
Δρ_min_ = −0.39 e Å^−3^

### 

Data collection: *APEX2* (Bruker, 2007 ▶); cell refinement: *SAINT* (Bruker, 2007 ▶); data reduction: *SAINT*; program(s) used to solve structure: *SHELXS97* (Sheldrick, 2008 ▶); program(s) used to refine structure: *SHELXL97* (Sheldrick, 2008 ▶); molecular graphics: *ORTEP-3 for Windows* (Farrugia, 1997 ▶) and *PLATON* (Spek, 2009 ▶); software used to prepare material for publication: *WinGX* (Farrugia, 1999 ▶) and *PLATON*.

## Supplementary Material

Crystal structure: contains datablock(s) global, I. DOI: 10.1107/S1600536812015413/su2404sup1.cif

Structure factors: contains datablock(s) I. DOI: 10.1107/S1600536812015413/su2404Isup2.hkl

Supplementary material file. DOI: 10.1107/S1600536812015413/su2404Isup3.cml

Additional supplementary materials:  crystallographic information; 3D view; checkCIF report

## Figures and Tables

**Table 1:** Hydrogen-bond geometry (Å, °) *Cg*1 is the centroid of the C2–C7 benzene ring.

*D*—H⋯*A*	*D*—H	H⋯*A*	*D*⋯*A*	*D*—H⋯*A*
N1—H1⋯O3^i^	0.86	2.11	2.868 (4)	146
C9—H9⋯O2	0.93	2.36	3.015 (4)	127
C10—H10⋯O1^ii^	0.93	2.53	3.453 (4)	173
C1—H1*C*⋯*Cg*1^iii^	0.96	2.76	3.639 (6)	153

Symmetry codes: (i) 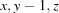; (ii) 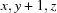; (iii) 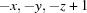.

**Fig. 1.**
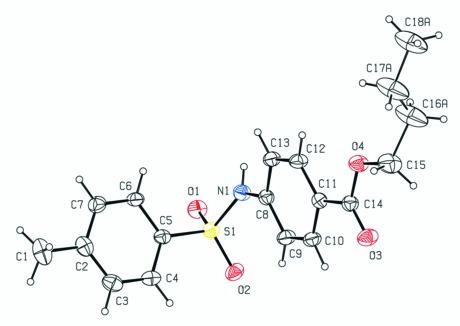
The molecular structure of the title molecule, with the atom numbering. Displacement ellipsoids are drawn at the 20% probability level. Only the atoms of the major disordered component of the butyl group are shown.

**Fig. 2.**
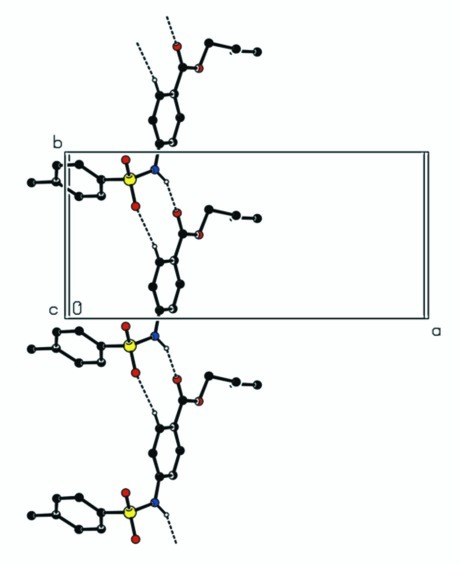
A partial view along the c axis of the crystal packing of the title compound. The N—H···O and C—H···O hydrogen bonds are shown as dashed lines (see Table 1 for details). Only the atoms of the major disordered component of the terminal butyl group are shown.

## supplementary crystallographic information

### Comment

As part of our ongoing studies of sulfonamides with potential biological properties (Mustafa *et al.*, 2010, 2011, 2012; Khan *et al.*, 2011), we describe herein the synthesis and crystal structure of the title compound.

As seen in Fig. 1, the two aromatic rings (C2—C7) and (C8—C13) are almost normal to each other, with a dihedral angle of 89.27 (18)°. The S atom has a distorted tetrahedral coordination geometry [S1—O1 = 1.411 (3), S1—O2 = 1.419 (3), S1— N1 = 1.626 (3), S1—C5 = 1.760 (4) Å, O1—S1—O2 = 120.43 (15), O1—S1—N1 = 105.23 (17), O1—S1 —C5 = 106.92 (16), O2—S1—N1 = 109.21 (16), O2—S1—C5 = 108.23 (17) and N1— S1—C5 = 105.94 (15)°]. All the bond lengths (Allen *et al.*, 1987) and angles are within normal ranges and are comparable to those found for similar structures (Mustafa *et al.*, 2010, 2011, 2012; Khan *et al.*, 2011).

The molecular conformation of the title compound is stabilized by an intramolecular C—H···O interaction, generating an S(6) motif (Table 1; Bernstein *et al.*, 1995). In the crystal, N—H···O and C—H···O hydrogen bonds lead to the formation of chains propagating along [010] - see Fig. 2 and Table 1. Neighbouring chains are linked *via* a C—H···π interaction (Table 1).

### Experimental

To an aquious solution of *p*-amino benzoic acid (1.0 g, 7.3 mmol), sodium carbonate (1 N) was added to adjust the pH to 8. *p*-toluenesulfonyl chloride (1.80 g, 9.48 mmol) was then added and the mixture was stirred at room temperature keeping the pH of the mixture up to 8 with occasional addition of sodium carbonate solution. The progress and completion of the reaction was confirmed by TLC and conversion of the suspension into a clear solution. After 2 h, the mixture was poured into a beaker and the pH was adjusted to 2.0 by addition of 1 N HCl. Precipitates were produced which were filtered and washed with distilled water. The prepared sulfonamide (4-(toluene-4-sulfonylamino)-benzoic acid) (1.0 g, 3.43 mmol), DMF (10 ml) and n-hexane washed with sodium hydride (0.25 g, 10.31 mmol) were stirred at room temperature for 40 min, followed by the addition of butyl iodide (0.94 g, 5.15 mmol). The whole reaction mixture was stirred till the completion of the reaction and poured into crushed ice in a beaker. The pH of the mixture was adjusted to 4.0 with 1 N HCl. Precipitates were produced, filtered and washed twice with distilled water. Crystallization in chloroform gave long block-like pale-yellow X-ray quality crystals of the title compound.

### Refinement

All the H-atoms were included in calculated positions and treated as riding atoms: N—H = 0.88 (2) Å, C—H = 0.93, 0.96 and 0.97 Å for CH, CH_3_ and CH_2_ H-atoms, respectively, with *U*_iso_(H) = k × *U*_eq_(N,*C*), where k = 1.5 for CH_3_ H-atoms and = 1.2 for all other H-atoms. The –CH_2_—CH_2_—CH_3_ atoms (C16, C17 and C18) of the butyl group are disordered over two sets of sites (A/B), with a refined site occupancy ratio of 0.536 (16):0.464 (16). Twelve poorly fitted reflections (1 0 0), (1 1 2), (-1 4 2), (1 3 4), (0 2 2), (-11 3 6), (8 3 0), (6 1 2), (2 3 8), (11 1 0), (-7 2 2) and (-14 3 4) were omitted from the refinement.

### Crystal data

**Table:**

C_18_H_21_NO_4_S	*F*(000) = 736
*M**_r_* = 347.43	*D*_x_ = 1.313 Mg m^−^^3^
Monoclinic, *P*2_1_/*c*	Mo *K*α radiation, λ = 0.71073 Å
Hall symbol: -P 2ybc	Cell parameters from 2942 reflections
*a* = 17.8216 (13) Å	θ = 2.7–21.5°
*b* = 8.2702 (6) Å	µ = 0.21 mm^−^^1^
*c* = 11.9282 (8) Å	*T* = 296 K
β = 91.001 (3)°	Long block, light yellow
*V* = 1757.8 (2) Å^3^	0.33 × 0.25 × 0.21 mm
*Z* = 4	

### Data collection

**Table:**

Bruker APEXII CCD diffractometer	2287 reflections with *I* > 2σ(*I*)
Radiation source: sealed tube	*R*_int_ = 0.038
Graphite monochromator	θ_max_ = 26.4°, θ_min_ = 3.0°
φ and ω scans	*h* = −21→22
13164 measured reflections	*k* = −10→10
3557 independent reflections	*l* = −11→14

### Refinement

**Table:**

Refinement on *F*^2^	Primary atom site location: structure-invariant direct methods
Least-squares matrix: full	Secondary atom site location: difference Fourier map
*R*[*F*^2^ > 2σ(*F*^2^)] = 0.072	Hydrogen site location: inferred from neighbouring sites
*wR*(*F*^2^) = 0.218	H-atom parameters constrained
*S* = 1.05	*w* = 1/[σ^2^(*F*_o_^2^) + (0.1157*P*)^2^ + 0.8149*P*] where *P* = (*F*_o_^2^ + 2*F*_c_^2^)/3
3557 reflections	(Δ/σ)_max_ < 0.001
224 parameters	Δρ_max_ = 0.47 e Å^−^^3^
0 restraints	Δρ_min_ = −0.39 e Å^−^^3^

### Special details

**Table:**

Geometry. Bond distances, angles *etc*. have been calculated using the rounded fractional coordinates. All su's are estimated from the variances of the (full) variance-covariance matrix. The cell e.s.d.'s are taken into account in the estimation of distances, angles and torsion angles
Refinement. Refinement on *F*^2^ for ALL reflections except those flagged by the user for potential systematic errors. Weighted *R*-factors *wR* and all goodnesses of fit *S* are based on *F*^2^, conventional *R*-factors *R* are based on *F*, with *F* set to zero for negative *F*^2^. The observed criterion of *F*^2^ > σ(*F*^2^) is used only for calculating -*R*-factor-obs *etc*. and is not relevant to the choice of reflections for refinement. *R*-factors based on *F*^2^ are statistically about twice as large as those based on *F*, and *R*-factors based on ALL data will be even larger.

### Fractional atomic coordinates and isotropic or equivalent isotropic displacement parameters (Å^2^)

**Table:**

	*x*	*y*	*z*	*U*_iso_*/*U*_eq_	Occ. (<1)
S1	0.17821 (5)	−0.16383 (11)	0.79992 (7)	0.0561 (3)	
O1	0.19456 (16)	−0.3261 (3)	0.8276 (2)	0.0672 (9)	
O2	0.16764 (16)	−0.0474 (3)	0.88556 (19)	0.0707 (10)	
O3	0.30722 (17)	0.6342 (3)	0.5914 (2)	0.0779 (10)	
O4	0.36618 (16)	0.5025 (3)	0.4580 (2)	0.0732 (10)	
N1	0.24647 (17)	−0.1059 (4)	0.7206 (2)	0.0618 (10)	
C1	−0.0991 (3)	−0.1840 (7)	0.5042 (4)	0.0992 (19)	
C2	−0.0300 (2)	−0.1785 (5)	0.5775 (3)	0.0666 (14)	
C3	−0.0269 (2)	−0.0810 (5)	0.6726 (4)	0.0725 (17)	
C4	0.0361 (2)	−0.0747 (5)	0.7391 (3)	0.0618 (12)	
C5	0.09731 (19)	−0.1661 (4)	0.7132 (3)	0.0506 (10)	
C6	0.0964 (2)	−0.2636 (4)	0.6183 (3)	0.0582 (12)	
C7	0.0334 (2)	−0.2668 (5)	0.5514 (3)	0.0659 (16)	
C8	0.25941 (18)	0.0499 (4)	0.6752 (3)	0.0515 (11)	
C9	0.2412 (2)	0.1907 (4)	0.7308 (3)	0.0641 (14)	
C10	0.2598 (2)	0.3388 (4)	0.6868 (3)	0.0625 (12)	
C11	0.29879 (18)	0.3488 (4)	0.5867 (3)	0.0528 (11)	
C12	0.3148 (2)	0.2070 (5)	0.5310 (3)	0.0618 (12)	
C13	0.2953 (2)	0.0585 (4)	0.5737 (3)	0.0582 (12)	
C14	0.3224 (2)	0.5084 (4)	0.5476 (3)	0.0573 (12)	
C15	0.3938 (3)	0.6546 (6)	0.4172 (5)	0.102 (2)	
C16A	0.4688 (9)	0.619 (2)	0.3570 (12)	0.152 (5)	0.536 (16)
C17A	0.4608 (8)	0.607 (2)	0.2481 (12)	0.152 (5)	0.536 (16)
C18A	0.5254 (9)	0.601 (2)	0.1612 (13)	0.152 (5)	0.536 (16)
C17B	0.5053 (7)	0.5832 (16)	0.3103 (10)	0.086 (3)	0.464 (16)
C18B	0.5446 (7)	0.5739 (17)	0.2028 (10)	0.086 (3)	0.464 (16)
C16B	0.4245 (7)	0.6307 (15)	0.2980 (10)	0.086 (3)	0.464 (16)
H1B	−0.10320	−0.28850	0.46960	0.1490*	
H3	−0.06840	−0.01930	0.69120	0.0870*	
H4	0.03750	−0.00810	0.80190	0.0740*	
H1C	−0.09590	−0.10240	0.44720	0.1490*	
H1	0.27880	−0.17900	0.70400	0.0740*	
H1A	−0.14250	−0.16450	0.54880	0.1490*	
H10	0.24620	0.43290	0.72400	0.0750*	
H12	0.33930	0.21190	0.46280	0.0740*	
H13	0.30630	−0.03550	0.53440	0.0700*	
H15B	0.40420	0.72770	0.47910	0.1220*	0.536 (16)
H15D	0.35690	0.70440	0.36740	0.1220*	0.536 (16)
H16C	0.50410	0.70580	0.37400	0.1820*	0.536 (16)
H16D	0.49000	0.51960	0.38630	0.1820*	0.536 (16)
H17C	0.42890	0.69650	0.22530	0.1820*	0.536 (16)
H17D	0.43160	0.50960	0.23530	0.1820*	0.536 (16)
H18D	0.57300	0.60340	0.20010	0.2280*	0.536 (16)
H18E	0.52150	0.69330	0.11240	0.2280*	0.536 (16)
H18F	0.52130	0.50390	0.11780	0.2280*	0.536 (16)
H6	0.13810	−0.32570	0.60060	0.0700*	
H7	0.03300	−0.32990	0.48680	0.0790*	
H9	0.21610	0.18520	0.79840	0.0770*	
H15A	0.43340	0.69440	0.46680	0.1220*	0.464 (16)
H15C	0.35360	0.73360	0.41540	0.1220*	0.464 (16)
H16A	0.39650	0.54670	0.25890	0.1030*	0.464 (16)
H16B	0.42000	0.73020	0.25530	0.1030*	0.464 (16)
H17A	0.53080	0.66120	0.35830	0.1030*	0.464 (16)
H17B	0.50830	0.47870	0.34710	0.1030*	0.464 (16)
H18A	0.50870	0.55730	0.14300	0.1280*	0.464 (16)
H18B	0.57940	0.48530	0.20490	0.1280*	0.464 (16)
H18C	0.57130	0.67290	0.19040	0.1280*	0.464 (16)

### Atomic displacement parameters (Å^2^)

**Table:**

	*U*^11^	*U*^22^	*U*^33^	*U*^12^	*U*^13^	*U*^23^
S1	0.0664 (6)	0.0498 (5)	0.0523 (5)	−0.0032 (4)	0.0105 (4)	0.0025 (4)
O1	0.0839 (17)	0.0506 (15)	0.0674 (15)	−0.0016 (13)	0.0124 (13)	0.0131 (12)
O2	0.0923 (19)	0.0690 (17)	0.0512 (13)	−0.0142 (14)	0.0126 (13)	−0.0087 (12)
O3	0.102 (2)	0.0505 (16)	0.0821 (18)	−0.0024 (14)	0.0267 (16)	−0.0034 (14)
O4	0.0831 (18)	0.0614 (17)	0.0760 (17)	−0.0054 (13)	0.0292 (15)	0.0018 (13)
N1	0.0649 (18)	0.0483 (16)	0.0727 (19)	−0.0003 (14)	0.0159 (15)	0.0005 (14)
C1	0.079 (3)	0.122 (4)	0.096 (3)	−0.023 (3)	−0.016 (3)	0.043 (3)
C2	0.065 (2)	0.071 (3)	0.064 (2)	−0.010 (2)	0.0044 (18)	0.026 (2)
C3	0.069 (3)	0.069 (3)	0.080 (3)	0.014 (2)	0.014 (2)	0.013 (2)
C4	0.073 (2)	0.056 (2)	0.057 (2)	0.0118 (18)	0.0151 (18)	−0.0002 (17)
C5	0.0594 (19)	0.0450 (18)	0.0479 (17)	−0.0002 (15)	0.0139 (15)	0.0061 (14)
C6	0.061 (2)	0.060 (2)	0.054 (2)	0.0045 (17)	0.0137 (17)	−0.0102 (16)
C7	0.075 (3)	0.073 (3)	0.050 (2)	−0.005 (2)	0.0067 (19)	−0.0060 (18)
C8	0.0508 (18)	0.0503 (19)	0.0536 (18)	−0.0025 (15)	0.0047 (15)	0.0010 (15)
C9	0.078 (3)	0.054 (2)	0.061 (2)	−0.0025 (18)	0.0210 (19)	−0.0067 (17)
C10	0.070 (2)	0.052 (2)	0.066 (2)	0.0026 (17)	0.0158 (18)	−0.0109 (17)
C11	0.0501 (18)	0.054 (2)	0.0545 (18)	−0.0032 (15)	0.0042 (15)	−0.0006 (16)
C12	0.071 (2)	0.061 (2)	0.054 (2)	0.0027 (18)	0.0191 (17)	−0.0031 (17)
C13	0.066 (2)	0.050 (2)	0.059 (2)	−0.0004 (17)	0.0144 (17)	−0.0052 (16)
C14	0.057 (2)	0.054 (2)	0.061 (2)	0.0013 (16)	0.0057 (17)	−0.0010 (17)
C15	0.118 (4)	0.074 (3)	0.115 (4)	−0.012 (3)	0.056 (3)	0.015 (3)
C16A	0.107 (7)	0.237 (12)	0.113 (7)	0.056 (6)	0.042 (5)	0.048 (6)
C17A	0.107 (7)	0.237 (12)	0.113 (7)	0.056 (6)	0.042 (5)	0.048 (6)
C18A	0.107 (7)	0.237 (12)	0.113 (7)	0.056 (6)	0.042 (5)	0.048 (6)
C17B	0.062 (5)	0.104 (5)	0.091 (6)	−0.005 (3)	0.007 (3)	0.015 (4)
C18B	0.062 (5)	0.104 (5)	0.091 (6)	−0.005 (3)	0.007 (3)	0.015 (4)
C16B	0.062 (5)	0.104 (5)	0.091 (6)	−0.005 (3)	0.007 (3)	0.015 (4)

### Geometric parameters (Å, º)

**Table:**

S1—O1	1.411 (3)	C1—H1A	0.9600
S1—O2	1.419 (3)	C1—H1B	0.9600
S1—N1	1.626 (3)	C1—H1C	0.9600
S1—C5	1.760 (4)	C3—H3	0.9300
O3—C14	1.197 (4)	C4—H4	0.9300
O4—C14	1.335 (4)	C6—H6	0.9300
O4—C15	1.439 (6)	C7—H7	0.9300
N1—C8	1.418 (5)	C9—H9	0.9300
N1—H1	0.8600	C10—H10	0.9300
C1—C2	1.499 (6)	C12—H12	0.9300
C2—C3	1.392 (6)	C13—H13	0.9300
C2—C7	1.385 (5)	C15—H15B	0.9700
C3—C4	1.364 (5)	C15—H15D	0.9700
C4—C5	1.367 (5)	C15—H15A	0.9700
C5—C6	1.390 (5)	C15—H15C	0.9700
C6—C7	1.366 (5)	C16A—H16C	0.9700
C8—C13	1.381 (5)	C16A—H16D	0.9700
C8—C9	1.382 (5)	C16B—H16B	0.9700
C9—C10	1.375 (5)	C16B—H16A	0.9700
C10—C11	1.394 (5)	C17A—H17D	0.9700
C11—C12	1.380 (5)	C17A—H17C	0.9700
C11—C14	1.464 (5)	C17B—H17A	0.9700
C12—C13	1.377 (5)	C17B—H17B	0.9700
C15—C16B	1.545 (13)	C18A—H18E	0.9600
C15—C16A	1.556 (17)	C18A—H18F	0.9600
C16A—C17A	1.31 (2)	C18A—H18D	0.9600
C16B—C17B	1.497 (18)	C18B—H18A	0.9600
C17A—C18A	1.56 (2)	C18B—H18B	0.9600
C17B—C18B	1.474 (17)	C18B—H18C	0.9600
			
O1—S1—O2	120.43 (15)	C6—C7—H7	119.00
O1—S1—N1	105.23 (17)	C8—C9—H9	120.00
O1—S1—C5	106.92 (16)	C10—C9—H9	120.00
O2—S1—N1	109.21 (16)	C9—C10—H10	120.00
O2—S1—C5	108.23 (17)	C11—C10—H10	120.00
N1—S1—C5	105.94 (15)	C11—C12—H12	119.00
C14—O4—C15	116.6 (3)	C13—C12—H12	119.00
S1—N1—C8	128.2 (3)	C8—C13—H13	120.00
C8—N1—H1	116.00	C12—C13—H13	120.00
S1—N1—H1	116.00	O4—C15—H15B	110.00
C3—C2—C7	117.9 (3)	O4—C15—H15D	110.00
C1—C2—C7	121.2 (4)	O4—C15—H15A	110.00
C1—C2—C3	120.9 (4)	O4—C15—H15C	110.00
C2—C3—C4	121.1 (4)	C16A—C15—H15B	108.00
C3—C4—C5	120.0 (4)	C16A—C15—H15D	112.00
S1—C5—C6	118.9 (3)	H15B—C15—H15D	109.00
S1—C5—C4	120.7 (3)	C16B—C15—H15A	110.00
C4—C5—C6	120.5 (3)	C16B—C15—H15C	110.00
C5—C6—C7	119.0 (3)	H15A—C15—H15C	108.00
C2—C7—C6	121.6 (3)	C15—C16A—H16C	109.00
N1—C8—C9	122.8 (3)	C15—C16A—H16D	109.00
N1—C8—C13	117.6 (3)	C17A—C16A—H16C	109.00
C9—C8—C13	119.6 (3)	C17A—C16A—H16D	109.00
C8—C9—C10	120.5 (3)	H16C—C16A—H16D	108.00
C9—C10—C11	120.4 (3)	H16A—C16B—H16B	109.00
C12—C11—C14	123.4 (3)	C15—C16B—H16A	110.00
C10—C11—C14	118.5 (3)	C15—C16B—H16B	110.00
C10—C11—C12	118.2 (3)	C17B—C16B—H16A	110.00
C11—C12—C13	121.6 (3)	C17B—C16B—H16B	110.00
C8—C13—C12	119.6 (3)	C18A—C17A—H17C	106.00
O3—C14—C11	125.2 (3)	C18A—C17A—H17D	106.00
O3—C14—O4	121.4 (3)	C16A—C17A—H17D	106.00
O4—C14—C11	113.4 (3)	C16A—C17A—H17C	106.00
O4—C15—C16B	109.2 (6)	H17C—C17A—H17D	106.00
O4—C15—C16A	107.0 (7)	C16B—C17B—H17A	109.00
C15—C16A—C17A	113.1 (12)	C16B—C17B—H17B	109.00
C15—C16B—C17B	107.4 (9)	H17A—C17B—H17B	108.00
C16A—C17A—C18A	126.3 (14)	C18B—C17B—H17B	109.00
C16B—C17B—C18B	113.5 (10)	C18B—C17B—H17A	109.00
C2—C1—H1A	109.00	C17A—C18A—H18F	110.00
C2—C1—H1B	109.00	C17A—C18A—H18E	109.00
C2—C1—H1C	109.00	H18E—C18A—H18F	110.00
H1A—C1—H1B	109.00	H18D—C18A—H18E	109.00
H1A—C1—H1C	109.00	H18D—C18A—H18F	110.00
H1B—C1—H1C	109.00	C17A—C18A—H18D	109.00
C2—C3—H3	120.00	C17B—C18B—H18A	109.00
C4—C3—H3	119.00	C17B—C18B—H18B	109.00
C3—C4—H4	120.00	C17B—C18B—H18C	110.00
C5—C4—H4	120.00	H18A—C18B—H18B	109.00
C5—C6—H6	120.00	H18A—C18B—H18C	109.00
C7—C6—H6	121.00	H18B—C18B—H18C	109.00
C2—C7—H7	119.00		
			
O1—S1—N1—C8	−176.1 (3)	C3—C4—C5—C6	1.1 (6)
O2—S1—N1—C8	−45.5 (3)	S1—C5—C6—C7	179.4 (3)
C5—S1—N1—C8	70.9 (3)	C4—C5—C6—C7	−0.1 (5)
O1—S1—C5—C4	126.3 (3)	C5—C6—C7—C2	−1.6 (6)
O1—S1—C5—C6	−53.2 (3)	N1—C8—C9—C10	175.3 (3)
O2—S1—C5—C4	−4.8 (4)	C13—C8—C9—C10	−1.2 (5)
O2—S1—C5—C6	175.7 (3)	N1—C8—C13—C12	−174.5 (3)
N1—S1—C5—C4	−121.9 (3)	C9—C8—C13—C12	2.2 (5)
N1—S1—C5—C6	58.7 (3)	C8—C9—C10—C11	−1.4 (5)
C15—O4—C14—O3	−0.1 (5)	C9—C10—C11—C12	3.0 (5)
C15—O4—C14—C11	−177.9 (3)	C9—C10—C11—C14	−175.4 (3)
C14—O4—C15—C16A	153.3 (6)	C10—C11—C12—C13	−2.0 (5)
S1—N1—C8—C9	33.7 (5)	C14—C11—C12—C13	176.3 (3)
S1—N1—C8—C13	−149.7 (3)	C10—C11—C14—O3	−4.6 (5)
C1—C2—C3—C4	−179.5 (4)	C10—C11—C14—O4	173.1 (3)
C7—C2—C3—C4	−1.1 (6)	C12—C11—C14—O3	177.1 (4)
C1—C2—C7—C6	−179.4 (4)	C12—C11—C14—O4	−5.2 (5)
C3—C2—C7—C6	2.2 (6)	C11—C12—C13—C8	−0.6 (5)
C2—C3—C4—C5	−0.5 (6)	O4—C15—C16A—C17A	98.0 (13)
C3—C4—C5—S1	−178.3 (3)	C15—C16A—C17A—C18A	169.1 (13)

### Hydrogen-bond geometry (Å, º)

Cg1 is the centroid of the C2–C7 benzene ring.

**Table:**

*D*—H···*A*	*D*—H	H···*A*	*D*···*A*	*D*—H···*A*
N1—H1···O3^i^	0.86	2.11	2.868 (4)	146
C4—H4···O2	0.93	2.53	2.908 (4)	105
C9—H9···O2	0.93	2.36	3.015 (4)	127
C10—H10···O1^ii^	0.93	2.53	3.453 (4)	173
C1—H1*C*···*Cg*1^iii^	0.96	2.76	3.639 (6)	153

Symmetry codes: (i) *x*, *y*−1, *z*; (ii) *x*, *y*+1, *z*; (iii) −*x*, −*y*, −*z*+1.

## References

[bb1] Allen, F. H., Kennard, O., Watson, D. G., Brammer, L., Orpen, A. G. & Taylor, R. (1987). *J. Chem. Soc. Perkin Trans. 2*, pp. S1–19.

[bb2] Bernstein, J., Davis, R. E., Shimoni, L. & Chang, N.-L. (1995). *Angew. Chem. Int. Ed. Engl.* **34**, 1555–1573.

[bb3] Bruker (2007). *APEX2* and *SAINT* Bruker AXS Inc., Madison, Wisconsin, USA.

[bb4] Farrugia, L. J. (1997). *J. Appl. Cryst.* **30**, 565.

[bb5] Farrugia, L. J. (1999). *J. Appl. Cryst.* **32**, 837–838.

[bb6] Khan, I. U., Mustafa, G. & Akkurt, M. (2011). *Acta Cryst.* E**67**, o1857. 10.1107/S1600536811025098 PMC3151892 21837222

[bb7] Mustafa, G., Akkurt, M., Khan, I. U., Naseem, R. & Sajjad, B. (2010). *Acta Cryst.* E**66**, o1768. 10.1107/S1600536810023925 PMC3006991 21587982

[bb8] Mustafa, G., Khan, I. U., Khan, F. M. & Akkurt, M. (2012). *Acta Cryst.* E**68**, o1305. 10.1107/S1600536812013864 PMC3344450 22590212

[bb9] Mustafa, G., Khan, I. U., Zia-ur-Rehman, M., Sharif, S. & Arshad, M. N. (2011). *Acta Cryst.* E**67**, o1018. 10.1107/S1600536811011524 PMC3099805 21754034

[bb10] Sheldrick, G. M. (2008). *Acta Cryst.* A**64**, 112–122. 10.1107/S0108767307043930 18156677

[bb11] Spek, A. L. (2009). *Acta Cryst.* D**65**, 148–155. 10.1107/S090744490804362X PMC2631630 19171970

